# ΔNp73 overexpression promotes resistance to apoptosis but does not cooperate with PML/RARA in the induction of an APL-leukemic phenotype

**DOI:** 10.18632/oncotarget.14295

**Published:** 2016-12-27

**Authors:** Antonio R. Lucena-Araujo, Juan L. Coelho-Silva, Diego A. Pereira-Martins, Carolina Thomé, Priscila S. Scheucher, Ana P. Lange, Helder H. Paiva, Benjamin T. Hemmelgarn, Mariana C. Morais-Sobral, Elisa A. Azevedo, Pedro L. Franca-Neto, Rafael F. Franca, Cleide L. Silva, Alexandre Krause, Eduardo M. Rego

**Affiliations:** ^1^ Department of Internal Medicine, Medical School of Ribeirao Preto, Brazil; ^2^ Department of Genetics, Federal University of Pernambuco, Recife, Brazil; ^3^ Center for Cell Based Therapy, University of Sao Paulo, Ribeirao Preto, Brazil; ^4^ The Ohio State University, Columbus, USA; ^5^ Department of Microbiology, Fundação Oswaldo Cruz, Centro de Pesquisas Aggeu Magalhães, Recife, Brazil; ^6^ Department of Virology, Fundação Oswaldo Cruz, Centro de Pesquisas Aggeu Magalhães, Recife, Brazil

**Keywords:** acute promyelocytic leukemia, ΔNp73, apoptosis, bone marrow transplantation, lentiviral gene transfer

## Abstract

Here, we evaluated whether the overexpression of transcriptionally inactive ΔNp73 cooperates with PML/RARA fusion protein in the induction of an APL-leukemic phenotype, as well as its role *in vitro* in proliferation, myeloid differentiation, and drug-induced apoptosis. Using lentiviral gene transfer, we showed *in vitro* that ΔNp73 overexpression resulted in increased proliferation in murine bone marrow (BM) cells from hCG-PML/RARA transgenic mice and their wild-type (WT) counterpart, with no accumulation of cells at G2/M or S phases; instead, ΔNp73-expressing cells had a lower rate of induced apoptosis. Next, we evaluated the effect of ΔNp73 on stem-cell self-renewal and myeloid differentiation. Primary BM cells lentivirally infected with human ΔNp73 were not immortalized in culture and did not present significant changes in the percentage of CD11b. Finally, we assessed the impact of ΔNp73 on leukemogenesis or its possible cooperation with PML/RARA fusion protein in the induction of an APL-leukemic phenotype. After 120 days of follow-up, all transplanted mice were clinically healthy and, no evidence of leukemia/myelodysplasia was apparent. Taken together, our data suggest that ΔNp73 had no leukemic transformation capacity by itself and apparently did not cooperate with the PML/RARA fusion protein to induce a leukemic phenotype in a murine BM transplantation model. In addition, the forced expression of ΔNp73 in murine BM progenitors did not alter the ATRA-induced differentiation rate *in vitro* or induce aberrant cell proliferation, but exerted an important role in cell survival, providing resistance to drug-induced apoptosis.

## INTRODUCTION

In the clinical setting, high expression of the NH2-terminal truncated ΔNp73 isoforms (alone or in association with full-length transcriptionally active TAp73, i.e., a high ΔNp73/TAp73 ratio) has been associated with poor prognosis in primary human tumors [1–3], including hematological malignancies [4–6]. Recently [7], we demonstrated that a high ΔNp73/TAp73 ratio is associated with inferior outcome in patients with acute promyelocytic leukemia (APL) treated with all-*trans* retinoic acid (ATRA) and anthracycline-based chemotherapy according to the International Consortium on APL 2005 protocol [8]. However, the mechanism through which ΔNp73 leads to adverse outcomes in APL remains to be elucidated and if ΔNp73 works as a driver oncogene in APL is unknown. Using lentiviral gene transfer and murine bone marrow (BM) transplantation, we evaluated whether the forced expression of ΔNp73 cooperates with PML/RARA fusion protein in the induction of an APL-leukemic phenotype. We also investigated the role of ΔNp73 in proliferation, myeloid differentiation, and drug-induced apoptosis.

## RESULTS AND DISCUSSION

**Figure 1:**
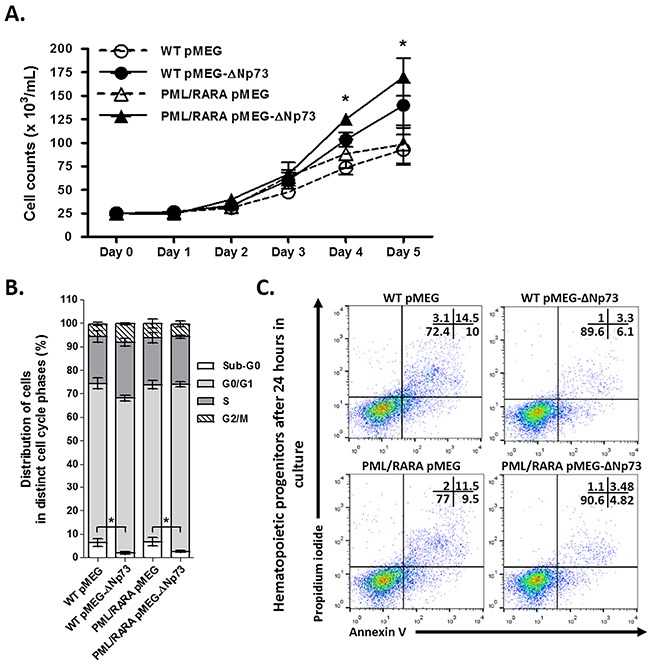
Characterization (in vitro assays) of primary hCG-PML/RARA-positive and WT hematopoietic progenitors infected with empty vector (pMEG) or pMEG-ΔNp73 lentiviruses Growth curves **A.** and subsequent cell cycle analysis **B.** in vitro of hCG-PML/RARA-positive and WT cells. Data are expressed as mean ± standard error of the mean **C.** Representative analysis of the number of apoptotic cells by Annexin-V/propidium iodide binding assay according to the presence or absence of ΔNp73. Primary BM cells from hCG-PML/RARA and WT mice were incubated with complete medium and no stimulus for apoptosis (spontaneous apoptosis) for 48-72 hours. * P < 0.05. Note: Comparison among all four groups were performed for proliferation assays using Kruskal-Wallis test with Dunn's multiple comparison post test. No significant differences was observed between WT pMEG and PML/RARA pMEG or WT pMEG-ΔNp73 and PML/RARA pMEG-ΔNp73 groups.

First, we evaluated the role of ΔNp73 in cell proliferation. At the end of the fifth day of culture, the forced expression of human ΔNp73 in hCG-PML/RARA and WT murine cells resulted in increased proliferation compared to respective controls (Figure 1A). Intriguingly, after subsequent cell cycle analysis, we found no accumulation of cells at G2/M or S phases. On the other hand, number of cells at sub-G0 fraction was significantly lower in ΔNp73-expressing cells than empty vector controls (Figure 1B). We subsequently validated these findings through annexin-V/propidium iodide staining method, demonstrating that the basal apoptosis rate (i.e., spontaneous apoptosis) was less pronounced in ΔNp73-expressing cells, regardless the presence of PML/RARA fusion gene (Figure 1C).

**Figure 2:**
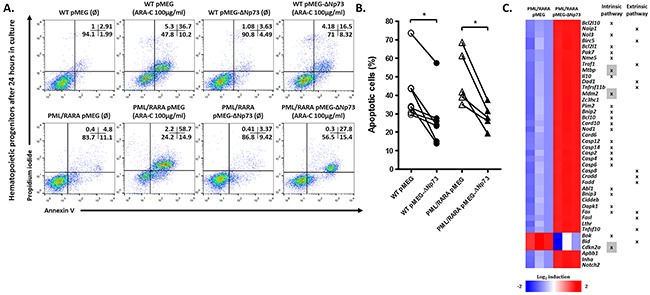
Drug-induced apoptosis assay **A.** Representative example of one out of five independent experiments of apoptosis using Ara-C 100μg/ml as standard stimulus for apoptosis. **B.** Percentage of apoptotic cells after 24h in culture after apoptotic stimulus. **C.** Fold induction upon Ara-C treatment of the top genes differentially expressed in hCG-PML-RARA cells overexpressing or not the ΔNp73. Genes related to intrinsic or extrinsic pathways are indicated. Genes highlighted in gray represent those related to cell cycle control. (Ø) represents non treated samples. * indicates P < 0.05.

These findings prompted us to investigate whether ΔNp73 overexpression results in resistance to drug-induced apoptosis. We performed an *in vitro* assay of apoptosis using cytarabine (Ara-C, IC50: 100μg/ml) as the apoptotic stimulus [9]. Twenty-four hours later, ΔNp73-expressing cells had a lower rate of apoptosis than empty vector controls (Figure 2A–2B). Next, we examined genes related to apoptosis and cell cycle pathways that were differentially expressed between hCG-PML/RARA cells (expressing or not the human ΔNp73 gene) upon Ara-C treatment. Using PCR array procedure, we identified a set of 42 genes (39 upregulated and three downregulated) differentially expressed in hCG-PML/RARA Np73-expressing cells in comparison with empty vector control (Figure 2C). As expected, most of genes were linked to apoptosis pathways. Only three genes related to cell cycle arrest were modulated by the presence of ΔNp73 in hCG-PM/RARA-positive cells (Mdm2, Cdkn2a (p21), Mtbp). Of interest, apoptosis-associated genes in Np73-expressing cells belonged to both mitochondrial-mediated (intrinsic pathway) and receptor-mediated pathways (extrinsic pathway).

**Figure 3:**
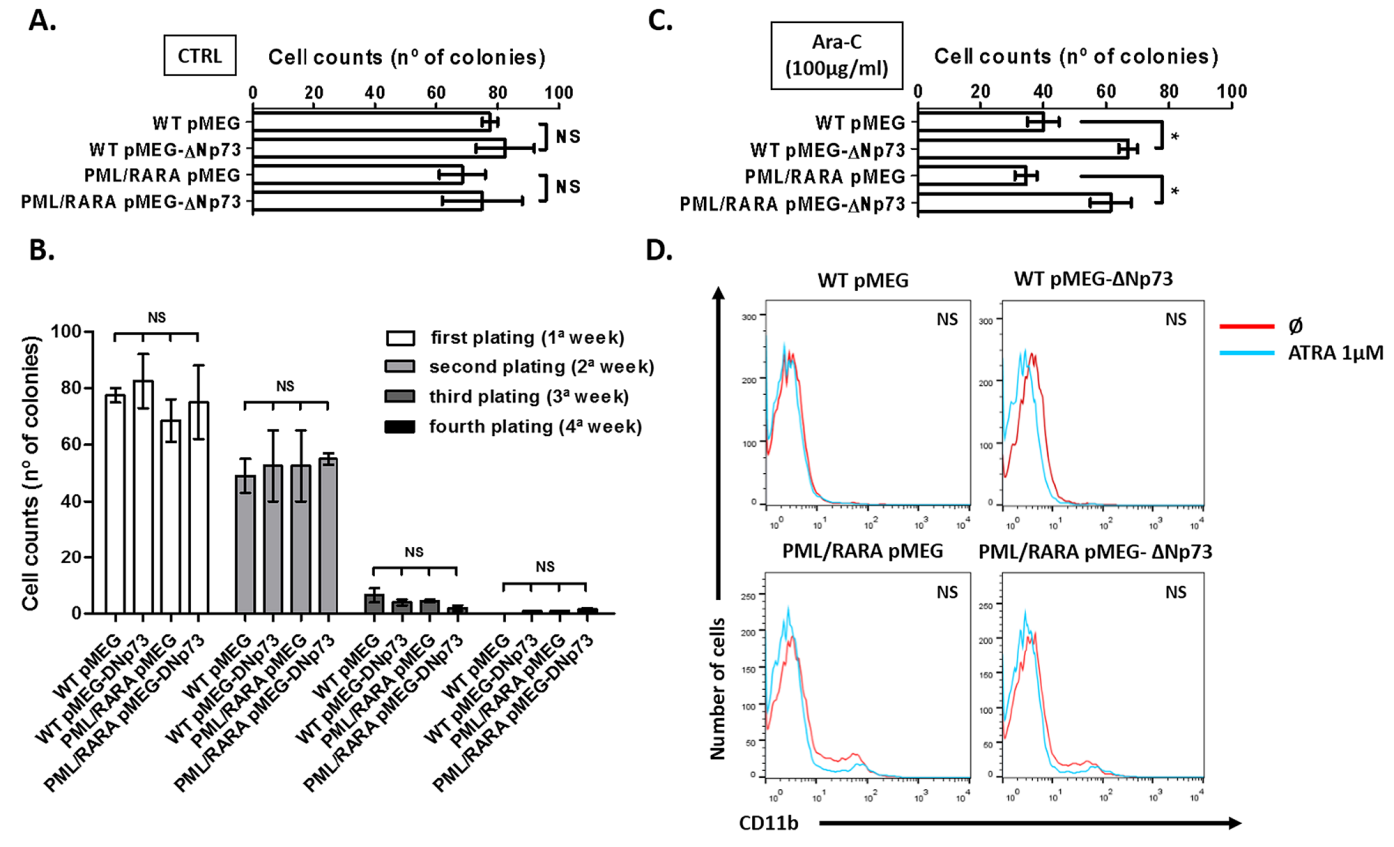
Effect of ΔNp73 on stem cell self-renewal and myeloid differentiation **A.** Number of colony-forming cells and **B.** clonogenic assay. Analysis of the number of colonies in methylcellulose. Bars represent the number of colonies counted after each plating. **C.** Colony forming assays performed in the presence of Ara-c 100 μg/ml. **D.** Myeloid differentiation. Percentage of CD11b-positive cells in hCG-PML/RARA cells or WT primary cells (infected with empty vector, pMEG or pMEG-ΔNp73 lentiviruses) using ATRA 1μM as the standard stimulus for differentiation. (Ø) represent non treated samples. * P < 0.05. NS: not significant. Note: Comparison among all four groups were performed using Kruskal-Wallis test with Dunn's multiple comparison post test. No significant differences was observed between WT pMEG and PML/RARA pMEG or WT pMEG-ΔNp73 and PML/RARA pMEG-ΔNp73 groups.

Next, we evaluated the effect of ΔNp73 on stem cell self-renewal and myeloid differentiation. Primary BM cells from hCG-PML/RARA and WT mice infected with human ΔNp73 were not immortalized in methylcellulose culture. At first plating, the final number of myeloid colonies was not different between ΔNp73-expressing cells and their respective controls (Figure 3A). In addition, the formation of colonies in methylcellulose was not sustained after the third to fourth plating in none of the groups (Figure 3B). Nevertheless, the final number of ΔNp73-expressing myeloid colonies was significantly higher compared to their respective controls after adding Ara-C 100 μg/ml to the methylcellulose cultures (Figure 3C), corroborating the resistance to drug-induced apoptosis observed in liquid culture. To check whether the forced expression of ΔNp73 affects myeloid differentiation, we evaluated the percentage of CD11b-positive cells using ATRA (1 μM, 7 days in liquid culture) as the standard stimulus for differentiation. Immunophenotypic analysis revealed that induced expression of human ΔNp73 did not significantly change the percentage of CD11b-positive cells (Figure 3D).

**Figure 4:**
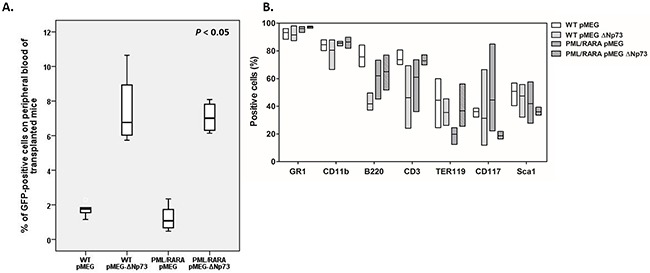
In vivo assays **A.** Percentage of GFP-positive cells in the peripheral blood of lethally irradiated recipient mice transplanted with PML/RARA-positive or WT bone marrow cells in the presence or absence of ΔNp73 overexpression. **B.** Immunophenotypic analysis of bone marrow recipients’ cells with respect to myeloid and lymphoid cell markers after transplantation. Box plots show the summarized data for immunophenotypic analyses of bone marrow from survival mice that received a transplant of empty vector control (WT, 7 animals; hCG-PML/RARA, 8 animals) or ΔNp73 (WT, 8 animals; hCG-PML/RARA, 6 animals). hGC-PML/RARA transplanted mice were not leukemic at the time of analysis. Bone marrow cells were stained for the indicated surface markers as indicated in the bottom of the figure. No significant difference between groups were detected.

Finally, we assessed the impact of ΔNp73 on leukemogenesis or its possible cooperation *in vivo* with PML/RARA fusion protein in the induction of an APL-leukemic phenotype by transplanting lethally irradiated NOD/SCID mice (10 animals per group) with 3×10^5^ GFP-positive BM cells (with or without human ΔNp73) from hCG-PML/RARA cells or WT mice, along with 2×10^5^ recipient-type BM cells. Following repopulation (at day 21 after transplantation), hemoglobin levels (P=0.03) and platelet counts (P=0.044) were significantly higher in both groups of mice transplanted with WT cells, regardless of the presence of ΔNp73 (Supplementary Table 1). Forty-five days after transplantation, a higher percentage of ΔNp73-expressing cells was detected in the peripheral blood of recipients compared to empty vector controls (P<0.05, Figure 4A). However, after 120 days of follow-up [10–12], all transplanted mice were clinically healthy and, according to the Bethesda criteria for hematopoietic neoplasms in mice [13], no evidence of leukemia or myelodysplasia was apparent after morphological evaluation of BM or spleen cells. Immunophenotypic analysis of BM recipients’ cells revealed no significant difference between ΔNp73-expressing and non-ΔNp73-expressing cells with respect to myeloid and lymphoid cell markers (Figure 4B).

The hypothesis that ΔNp73 harbors oncogenic potential is based on classical *in vitro* and *in vivo* transformation assays that demonstrated a proliferative advantage and/or cell immortalization of ΔNp73-expressing cells [14–16]. Other studies have suggested that differentiation, rather than proliferation and cell immortalization, dictates the oncogenic potential of ΔNp73 [17]. By delaying or blocking differentiation, ΔNp73 could prevent progenitors from reaching terminal differentiation, keeping these cells in a constant proliferative state and thereby enabling tumor development driven by cooperating oncogenes. Interestingly, ΔNp73 has been associated with the initiation of metastasis in melanoma cells [18] and several lines of evidence support ΔNp73 as an important determinant for apoptotic response to therapy [2, 9, 19]. Accordingly, cells from transgenic mice selectively deficient for the ΔNp73 isoforms have been shown to be more sensitive to DNA-damaging agents and undergo increased p53-dependent apoptosis [20]. On the other hand, the ectopic expression of transcriptionally active TAp73 was able to reduce drug resistance to chemotherapy in metastatic melanoma cells, which expressed high levels of ΔNp73 [21]. In agreement with this finding, down-regulation of the ΔNp73 isoforms by antisense techniques has been shown to enhance TP53/TAp73-mediated apoptosis in cancer cells in response to chemotherapy [22, 23].

Here, we did not see evidence of malignant transformation of pre-leukemic PML/RARA-positive cells by the forced expression of ΔNp73 in *in vitro* or *in vivo* experiments, but instead ΔNp73 overexpression provided an adaptive clonal advantage in the presence of a pro-apoptotic stimulus. One may argue that mice transplanted with PML/RARA-positive cells should develop an APL-leukemic phenotype; nevertheless, it worth to note that only 8-12% of hCG-PML/RARA transgenic mice develop leukemia [24], and several evidence have demonstrated that the presence of PML/RARA fusion oncoprotein in transgenic mice is not *per se* sufficient to cause leukemia [25–27]. Interestingly, the protective effect of ΔNp73 on cell death apparently was not influenced by the presence of the PML/RARA oncoprotein. Such an interaction would be expected, considering the intricate functional relationship between PML-p73-PML/RARA in APL blasts. At least when overexpressed, wild-type PML stabilizes p73 protein through acetylation, preventing its ubiquitination and subsequent degradation [28]. In APL, p73 stability/activity may be impaired mainly due to the disruption of PML-nuclear bodies by PML/RARA oncoprotein. Currently, whether distinct p73 isoforms (including the transactivation-deficient ΔNp73 isoform) are differentially affected by impaired PML functions is unknown.

Although the aforementioned and others issues remain to be elucidated, it is undeniable the role of the *TP53* family members and their intricate relationship in APL, in both onset and eradication of the disease. Gaillard et al. compared the gene expression and methylation profiles of purified promyelocyte populations from pre-leukemic MRP8-PML/RARA transgenic mice and showed that, in the absence of secondary lesions, PML/RARA has an overall limited impact on both the transcriptome and methylome [29]. The authors observed significant enrichment of the expression of cell cycle-related genes in PML/RARA promyelocytes, which led to expansion of the promyelocyte compartment and hypothesized that PML/RARA initiates leukemia by subtly shifting cell fate decisions within the promyelocyte compartment. Because our data suggest that ΔNp73 plays an important role in cell survival, it is conceivable that ΔNp73 participates in expansion of the promyelocytic compartment as an additional/secondary event. Following this reasoning, Ablain et al. [30] demonstrated that a functional Pml-p53 axis is required to eradicate leukemia-initiating cells in a mouse model of APL. Upon ATRA-induced PML/RARA degradation, normal Pml elicits nuclear bodies reformation and induces a p53 response, which exhibits features of senescence, but not apoptosis. According to our hypothesis, even with the restoration of the nuclear bodies upon ATRA treatment and subsequent activation of Trp53 signaling, it is theoretically possible that the overexpression of ΔNp73 protein impairs p53 tumor suppressor functions, and thus coffering survival advantage and/or resistance to chemotherapy to leukemic cells.

Taken together, these findings lead us to two major conclusions. First, ΔNp73 had no leukemic transformation capacity by itself and apparently did not cooperate with the PML/RARA fusion protein to induce a leukemic phenotype in a murine BM transplantation model. Second, the forced expression of ΔNp73 in murine BM progenitors did not alter the ATRA-induced differentiation rate *in vitro* or induce aberrant cell proliferation, but exerted an important role in cell survival, providing resistance to drug-induced apoptosis. We and others [2, 3, 6, 7, 19, 23, 31–34] support the idea that ΔNp73 overexpression may be an important determinant of the clinical response to chemotherapy and may offer a therapeutic target for enhancing chemosensitivity in human tumors, including APL. Of course, in the current scenario, the combination of ATRA and arsenic trioxide must be tested in ΔNp73-expressing cells in order to develop better strategies for patients who need special care, particularly those considered high-risk.

## MATERIALS AND METHODS

### Lentiviral transduction of primary murine bone marrow cells

#### Mice

Parental strain mice were bred and maintained at the Center for Cell Based Therapy Animal Facility. All experiments using mice were approved by the Institutional Animal Experimentation Ethics Committee (protocol number #088/2007) and conducted according to national guidelines for care and use of laboratory animals. Cathepsin G-PML/RARA (hCG-PML/RARA) transgenic mice [24] and their wild-type (WT) counterparts, at 8 to 12 weeks of age, were used as donors of primary BM for lentiviral infection and subsequent *in vitro* assays and BM transplantation. Female NOD/SCID mice, older than 8 weeks of age, were used as recipients for BM transplantation model. hCG-PML/RARA transgenic mice and WT littermates were kindly provided by Dr. Pier Paolo Pandolfi (Beth Israel Deaconess Medical Center, Harvard Stem Cell Institute, Boston, USA). All animals were housed under specific pathogen free conditions in individually ventilated cages during the whole experiment and were maintained according to the Guide for Care and Use of Laboratory Animals of the National Research Council, USA, and to the National Council of Animal Experiment Control recommendations.

#### Lentivirus production

Recombinant lentivirus encoding ΔNp73 gene was generated using pCDH1-MCS1-EF1-GFP-Puro (pMEG) (Supplementary Figure 1A) self-inactivating lentivector (#CD713B-1; System Biosciences, Mountain View, CA, USA) in 293T cells by following the three-plasmid packaging procedure as described elsewhere [35]. Briefly, the cDNA encoding hemagglutinin (HA)-tagged-ΔNp73 was subcloned into Xbal and BamHI sites downstream to murine stem cell virus (MSCV) promoter. The complete sequence of ΔNp73 gene was synthesized and fully sequenced in both directions by GenScript (Piscataway Township, NJ, USA). In addition to the MSCV promoter, the pMEG lentivector harbors the enhanced GFP (EGFP) gene and the puromycin resistance gene (PURO) under the control of the constitutive human elongation factor 1α (EF1) promoter. pMEG-lentivector (containing or not the encoding ΔNp73 gene; 22.5 μg/plate), pCMVΔR8.74 expressing HIV gag/pol, Rev and tat (15 μg/plate), and pMD2G vector expressing VSV-G (8 μg/plate) were transfected into five 100 mm culture dishes containing 293T cells using Lipofectamine 2000 (Invitrogen), according to manufacturer recommendations. Twenty-four hours later, the fresh lentiviral particle-containing supernatants were collected, filtered through 0.45-μm filters (Sarstedt, Nümbrecht, Germany) and used to infect the target cells.

#### Primary BM cell isolation and lentiviral infection

We used murine BM cells from healthy and age-matched hCG-PML/RARA transgenic mice and their WT counterparts as the target for lentiviral infection. Briefly, mice were treated with 150 mg/kg 5-fluorouracil for 5 days, sacrificed, and BM cells obtained by bone crushing. The cells were pre-stimulated for 48 hours in medium supplemented with 20% fetal bovine serum, murine interleukin-3 (6 ng/ml), murine interleukin-6 (10 ng/ml), and murine stem cell factor (100 ng/ml) in a humidified incubator at 37°C and 5% CO_2_. Next, the murine BM cells were infected with empty vector (pMEG) or pMEG-ΔNp73 lentiviruses for 4 to 6 hours, purified based on the expression of GFP protein (Supplementary Figure 1B), and posteriorly used for BM transplantation and *in vitro* assays.

### Western blotting and antibodies

Whole-cell lysates were prepared as previously described [36]. Mouse anti-β-actin (sc-81178) was purchased from Santa Cruz Biotechnology (Santa Cruz, CA, USA) and horseradish peroxidase-conjugated secondary antibody horse anti-mouse IgG (#7076) was purchased from Cell Signaling (Beverly, MA, USA). Anti-HA (NH2-terminal)-HRP Antibody (R930-25) was purchased from Life Technologies (Carlsbad, CA, USA). The antibody-protein complex was detected using the ECL Western Blotting Detection Reagents (GE Lifesciences Amersham, Buckinghamshire, Buckinghamshire, England).

### *In vitro* assays

#### Cell proliferation assay

Cell suspensions containing 1 × 10^5^ cells/mL of highly purified murine BM cells were seeded in triplicate in 25cm^2^ flask. Cell counts were performed daily for five days. Concomitantly, cell viability was determined by the Trypan blue dye exclusion method. Growth curves were drawn according to the number of cells/mL taking in account the final number of cells/incubation time. To calculate the cell proliferation only viable cells were considered. Three independent experiments were performed for each group of cells.

#### Cell cycle assay

For the cell cycle analysis, 1 × 10^6^ of highly purified murine BM cells were cultured in triplicate for 24 hours using complete medium in a 6-well plate and then were subjected to immunofluorescent staining of incorporated bromodeoxyuridine (BrdU) and 7-amino-actinomycin (7-AAD), followed by flow cytometric analysis using the BrdU Flow Kit (BD Biosciences, San Jose, CA, USA). Cells were incubated with 10μM of BrdU for 30 minutes, and then processed according to the manufacturer's recommendations.

#### Apoptosis assay

For the apoptosis analysis, 5 × 10^5^ of highly purified murine BM cells were incubated in complete medium for 24 hours in the presence of vehicle or Ara-C 100μg/ml. The apoptosis rate was determined using the Annexin V-APC and propidium iodide (PI) binding assay (BD Biosciences, San Jose, CA, USA), and analyzed by flow cytometry. All experiments were performed in triplicate and in each sample a minimum of 10 000 events were acquired in a FACSCalibur flow cytometer (BD Biosciences, San Jose, CA, USA).

#### Colony-forming cell (CFC) assay

CFCs were assayed in methylcellulose (Methocult M3434; STEMCELL Technologies Inc., Vancouver, Canada) supplemented with mIL-3 (6 ng/ml), mIL-6 (10 ng/ml), and mSCF (100 ng/ml), following the manufacturer's recommendations. One thousand viable cells/dish were plated in triplicate. Colonies were evaluated microscopically on day 8 after plating by using standard criteria.

#### Differentiation assay

Cell suspensions containing 5 × 10^5^ of highly purified murine BM cells were incubated in complete medium for seven days in the presence of ATRA (1μM). The differentiation rate was determined by immunophenotyping using the percentage of CD11b-positive cells (BD Biosciences, San Jose, CA, USA) as maturity marker. All experiments were performed in triplicate and in each sample a minimum of 10 000 events were acquired in a FACSCalibur flow cytometer (BD Biosciences, San Jose, CA, USA).

### PCR array

Both RT^2^ Profiler™ PCR Array Mouse Apoptosis™ and Mouse Cell Cycle (Qiagen, VA, USA) was employed to analyze the expression of a focused panel of genes in hCG-PML/RARA cells infected with empty vector (pMEG) or pMEG-ΔNp73 lentiviruses. Samples were run at the ABI 7500 (Applied Biosystems, CA, USA). The data analysis was performed in SDS software 2.3 (SABiosciences). Genes were analyzed using two-way hierarchical cluster analysis based on the relative expression (2^-ΔΔCt^) by Ward's method (JMP version 10.0.0, SAS Institute).

### Bone marrow transplantation

A total of 3 × 10^5^ of highly purified murine BM cells from WT mice and non-leukemic hCG-PML/RARA mice (containing or not ΔNp73) along with 2 × 10^5^ non-transfected cells of each background were injected into the lateral tail vein of lethally irradiated (3.5 Gy) NOD/SCID recipient mice. Recipients were aged older than 8 up to 12-weeks. During the whole experiment, all animals were maintained under specific pathogen-free environment in individually ventilated cages and observed for signs of disease. Sparfloxacin (10 μg/ml) was given into the drinking water for 2 to 3 weeks after BM transplantation. Viability of mice was monitored daily. Engraftment of donor cells was monitored by tail vein bleeds and FACS analysis of GFP positive cells was performed after 45 days.

### Flow cytometry

Freshly harvested BM cells from previously transplanted mice were suspended in phosphate-buffered saline contain 1% fetal bovine serum. The erythrocytes were lysed by alkaline lysis using AKC lysis buffer (0.15M NH4Cl, 1mM KHCO3, 0.1mM Na–ethylenediaminetetraacetic acid, pH: 7.4). The nucleated cells were then stained using fluorescence labeled antibodies to Gr1, CD11b, B220, CD3, Ter119, CD117, Sca1, or isotype controls (BD Biosciences, San Jose, CA, USA) following the manufacturer's recommendations. The flow cytometric data were collected using a BD FACSCalibur, and analyzed using CellQuest Pro software (BD Biosciences, San Jose, CA, USA).

### Statistical analysis

All calculations were performed using statistical package for the social sciences (SPSS) 19.0 software (IBM SPSS software, Chicago, IL, USA). All *P*-values were two sided with significance set to 5%.

## SUPPLEMENTARY DATA FIGURES AND TABLES


