# Supplementary material for: ΔNp73 overexpression promotes resistance to apoptosis but does not cooperate with PML/RARA in the induction of an APL-leukemic phenotype
Source: Oncotarget. 2016 Dec 27;8(5):8475–83. doi: 10.18632/oncotarget.14295 (PMC5352415; doi:10.18632/oncotarget.14295)
Supplement: Supplementary file 1 [file oncotarget-08-8475-s001.pdf]

ΔNp73 overexpression promotes resistance to apoptosis but does not cooperate with PML/RARA in the induction of an APL-leukemic phenotype

SUPPLEMENTARY DATA

MATERIALS AND METHODS

Cell line culture

The human APL derived cell line NB4 carrying the t(15;17) translocation and expressing the PML/RARA fusion protein was purchased from the American Tissue Culture Collection (ATCC, Rockville, MD) and was cultured at 37°C in a humidified atmosphere of 5% CO2 in air, in RPMI medium (GIBCOBRL, Grand Island, NY, USA), supplemented with 10% fetal bovine serum (FBS) (GIBCO-BRL, Grand Island, NY, USA), 20 mM Hepes. Cell viability was determined via trypan blue assay, and only cultures with 95% viability were used for subsequent *in vitro* experiments.

RESULTS

Generation of Δ*N*p73-PML/RARA double mutant murine cells

The human coding sequence of the ΔNp73 gene was stably overexpressed in murine hematopoietic BM cells from hCG-PML/RARA transgenic mice and its wild-type counterpart using a lentiviral vector in which ΔNp73 expression was driven by the MSCV promoter (Supplementary Figure 1A). To determine the efficiency of infection, GFP-positive cells were quantified by flow cytometry. Infected cells were highly purified based on the expression of GFP protein. Similar infection rates were reached in both hCG-PML/RARA and WT cells (Supplementary Figure 1B). Additionally, cytospin preparations stained with DAPI were used for fluorescence evaluation (Supplementary Figure 1C). In order to confirm if the infection of ΔNp73 gene had efficiently resulted in a translation of its protein, transfected murine BM cells were lysed and protein lysates prepared using previously described methods. As shown in Supplementary Figure 1D, cells infected with pMEG-ΔNp73 lentivirus efficiently expressed the ΔNp73 protein. Next, we took advantage of the PML/RARA-positive NB4 cell line to demonstrate the ratio between truncated (ΔNp73) and transcriptionally active (TAp73) isoforms in our lentiviral model (Supplementary Figure 2).

In vivo assays

Supplementary Table 1 shows the hematological parameters of recipient mice transplanted with PML/RARA-positive cells or WT cells, with or without human ΔNp73. After 120 days of follow-up, all recipients were censored and morphological evaluation of BM or spleen cells was performed. No evidence of malignant transformation or myelodysplasia was observed.

SUPPLEMENTARY FIGURES AND TABLE

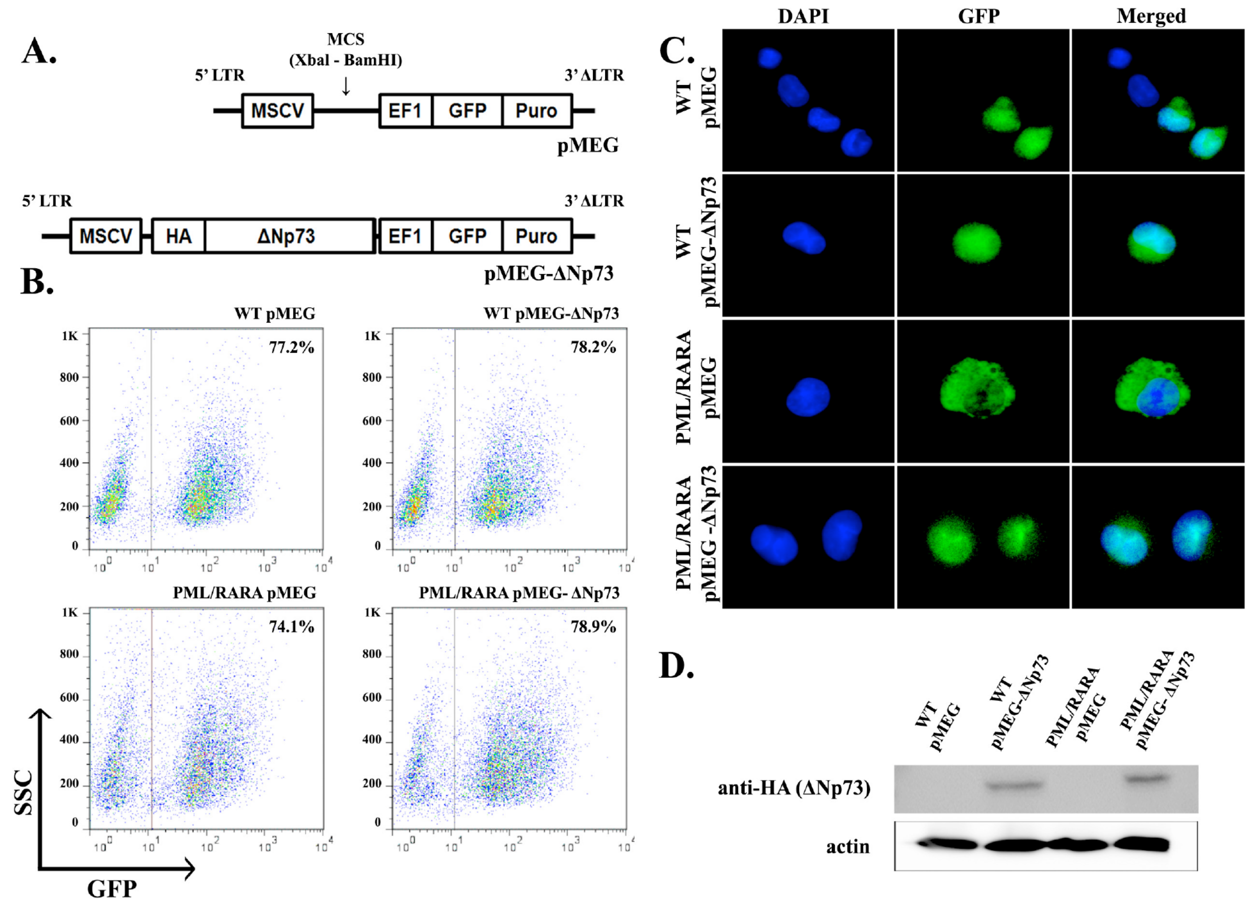

Supplementary Figure 1: A. Generation of ΔNp73-PML/RARA double mutant murine cells. Lentiviral vector encoding ΔNp73 cDNA. **B.** Representative FACS plot analysis of hCG-PML/RARA and WT murine BM cells overexpressing (GFP-positive) the ΔNp73 or not. **C.** Immunofluorescence analysis of cytospin preparations of BM cells stained with DAPI. **D.** Western blot of total protein lysates from PML/RARA-positive and WT cells infected with empty vector (pMEG) or pMEG-ΔNp73 lentiviruses. A total of 30 µg of protein was loaded in each lane.

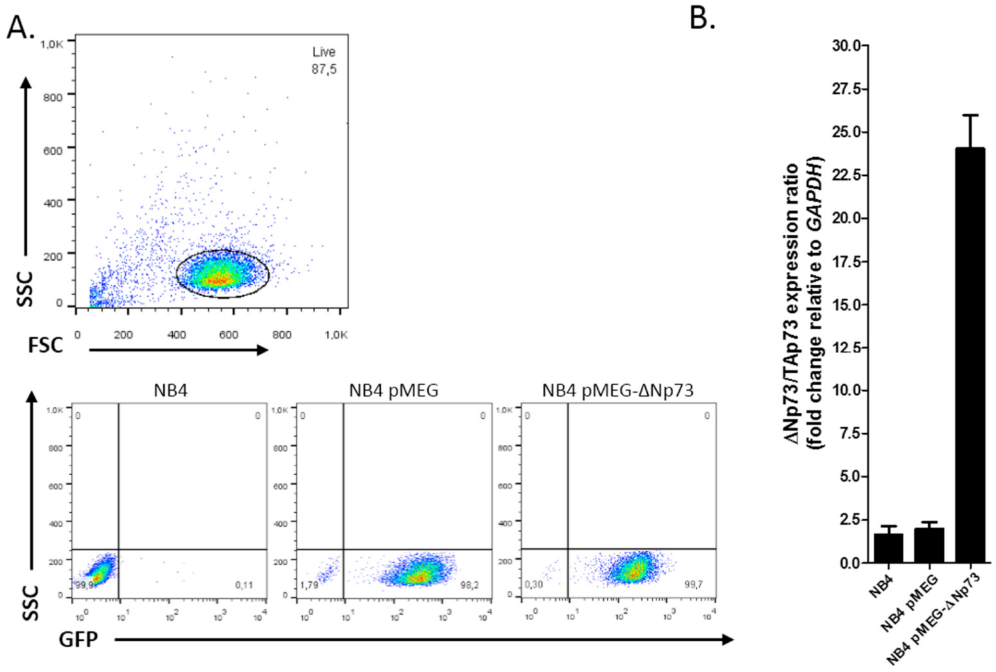

Supplementary Figure 2: ΔNp73/TAp73 expression ratio in NB4 cell line. A. Representative example of GFP-positive cells. After infection with pMEG or pMEG-ΔNp73 lentiviri, NB4 cell line was positive selected using sorting procedure. Percentage of GFP-positive cells are represented in plots. **B.** Real time quantitative polymerase chain reaction showing the upregulation of the ΔNp73. NB4 cell line infected with ΔNp73 lentivirus showed a 50-fold higher expression of ΔNp73/TAp73 ratio.

Supplementary Table 1: Hematological parameters of transplanted hCG-PML/RARA and WT mice

Groups	Hematological parameters		
	**Leucocytes (×10^9/L)**	**Hemoglobin (g/dl)**	**Platelets (×10^9/L)**
WT pMEG, n = 10	18 (11 to 23)	12 (10 to 14)	1430 (1180 to 1880)
WT pMEG-ΔNp73, n = 10	11 (9 to 19)	12.5 (9 to 14)	1155 (910 to 1250)
PML/RARA pMEG, n = 10	32 (9 to 201)	9 (7 to 10)	910 (610 to 1390)
PML/RARA pMEG-ΔNp73, n = 10	16 (7 to 19)	10 (9 to 13)	970 (850 to 1230)
P-value	0.34	0.03*	0.044*

The data were presented as median (range).
* *P*-value < 0.05 (one-way ANOVA followed by Bonferroni's multiple comparison test).
